# Excess mortality related to seasonal influenza and extreme temperatures in Denmark, 1994-2010

**DOI:** 10.1186/1471-2334-11-350

**Published:** 2011-12-16

**Authors:** Jens Nielsen, Anne Mazick, Steffen Glismann, Kåre Mølbak

**Affiliations:** 1 Statens Serum Institut, Department of Epidemiology, Artillerivej 5, DK2300 Copenhagen, Denmark

**Keywords:** influenza, mortality, temperature, seasonality

## Abstract

**Background:**

In temperate zones, all-cause mortality exhibits a marked seasonality, and one of the main causes of winter excess mortality is influenza. There is a tradition of using statistical models based on mortality from respiratory illnesses (Pneumonia and Influenza: PI) or all-cause mortality for estimating the number of deaths related to influenza. Different authors have applied different estimation methodologies. We estimated mortality related to influenza and periods with extreme temperatures in Denmark over the seasons 1994/95 to 2009/10.

**Methods:**

We applied a multivariable time-series model with all-cause mortality as outcome, activity of influenza-like illness (ILI) and excess temperatures as explanatory variables, controlling for trend, season, age, and gender. Two estimates of excess mortality related to influenza were obtained: (1) ILI-attributable mortality modelled directly on ILI-activity, and (2) influenza-associated mortality based on an influenza-index, designed to mimic the influenza transmission.

**Results:**

The median ILI-attributable mortality per 100,000 population was 35 (range 6 to 100) per season which corresponds to findings from comparable countries. Overall, 88% of these deaths occurred among persons ≥ 65 years of age. The median influenza-associated mortality per 100,000 population was 26 (range 0 to 73), slightly higher than estimates based on pneumonia and influenza cause-specific mortality as estimated from other countries. Further, there was a tendency of declining mortality over the years. The influenza A(H3N2) seasons of 1995/96 and 1998/99 stood out with a high mortality, whereas the A(H3N2) 2005/6 season and the 2009 A(H1N1) influenza pandemic had none or only modest impact on mortality. Variations in mortality were also related to extreme temperatures: cold winters periods and hot summers periods were associated with excess mortality.

**Conclusion:**

It is doable to model influenza-related mortality based on data on all-cause mortality and ILI, data that are easily obtainable in many countries and less subject to bias and subjective interpretation than cause-of-death data. Further work is needed to understand the variations in mortality observed across seasons and in particular the impact of vaccination against influenza.

## Background

In temperate zones, all-cause mortality exhibits a marked seasonality with the highest number of deaths in the winter and a lower number in the summer period. The reasons for this pattern are complex and not completely understood. Many factors may contribute, including increased rates of acute respiratory tract infections and death from cardio-vascular diseases in the winter months, periods with extreme temperature, and possibly mental and physiological effects (e.g. D-vitamin) related to day-light as well as social and psychological factors related to Christmas and New Year holidays [1]. However, it is well recognised that one of the main causes of winter excess mortality is influenza.

There is a long tradition of using statistical models based on mortality from respiratory illnesses (Pneumonia and Influenza: PI) or all-cause mortality for estimating the number of deaths related to influenza. Different authors have applied different estimation methodologies. The most commonly used methodology, often called Serflings method, estimate the expected mortality without influenza based on either predefined periods with no or ignorable influenza activity [2,3] or dynamically defined periods with no influenza activity classified according to influenza activity recorded under a certain level e.g. 95% confidence limit; data on influenza activity have been defined by influenza specific mortality [4-7], or Influenza Like Illness (ILI) reported from networks of sentinel practices [8-10] or reported as surveillance counts for laboratory virus-positive specimens [11,7]. Hence, periods with influenza-attributable mortality may vary from season to season according to each season's influenza activity. Excess mortality attributable to influenza is then calculated as the observed minus the modelled expected mortality without influenza as estimated from periods without influenza activity. Others has extended the estimation of the no-influenza expected mortality to exclude impact of other respiratory and circulatory diseases, like infections from Respiratory Syncytial Virus (RSV), by controlling for presence of these in the estimation of the expected mortality in the absence of influenza [9,11,12]. Instead of excluding periods with influenza activity from the estimation of the no-influenza expected mortality, these periods may be down-weighted [13]. Alternatively, influenza activity can be included directly as a parameter in a multivariable time-series model, then often using an identity-link, including influenza activity, trend and seasonal variation as independent variables [14,3,11,15]. Thus, in this latter approach excess mortality is determined directly from data on influenza and not as a residual difference.

Ambient temperature may also play a role in the seasonal variation of mortality [14,16-19], but this is often not included in assessment of influenza-related mortality.

The aim of the present study was to describe mortality associated to influenza and periods with extreme temperatures in Denmark over the seasons 1994/95 to 2009/10.

## Materials and methods

To investigate the impact of influenza and extreme temperatures on mortality we specified and applied a multivariable time-series model with all-cause mortality as outcome, influenza activity and excess temperatures as explanatory variables, and controlled for trend, season, age, and gender.

### Data sources

Data for the analyses were obtained from the following sources:

Individual notifications of deaths were obtained from the Danish civil registry system by the Department of Epidemiology, Statens Serum Institut (SSI), in the form of daily electronic notifications of all deaths.

The sentinel influenza surveillance system based on primary health care consultations was established in 1994 as a voluntary reporting system of general practitioners providing weekly reports on the total number of consultations and age-specific numbers of Influenza-Like-Illness (ILI) consultations to the Department of Epidemiology, Statens Serum Institut (SSI). This system is usually discontinued in the summer period between week 20 and 40, but in 2009 data were collected throughout the year.

**Figure 1:**
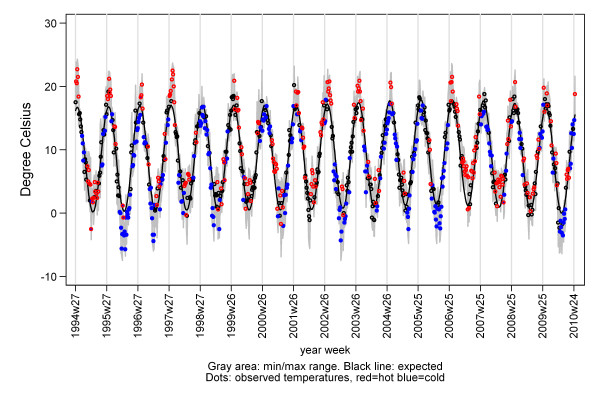
**Weekly Danish temperatures 1994/5 to 2009/10**.

Data on daily temperatures registered at Danish weather stations was downloaded from the National Oceanic and Atmospheric Administration Online Climate Data Directory [20]. Mean over daily temperatures from all weather stations was used as the overall Danish temperature for that day. Weekly temperatures were calculated as the mean over the week, as was the weekly minimum and maximum temperatures. Using the mean weekly temperatures, we estimated the expected weekly temperature in a General-Linear-Model with a sine seasonal variation (Figure 1). Weeks with extreme temperatures was defined by a threshold-linear model [21]. Warm weeks were defined as weeks with weekly minimum temperature > expected weekly temperature; cold weeks as weekly maximum temperature < expected weekly temperature and the effect as the linear difference between observed and minimum respectively maximum weekly temperature.

Size of the Danish population by age and gender on the 1^st ^of January every year was downloaded from Statistics Denmark [22] (Table 1). The weekly sizes of the age group and gender specific populations were achieved by linear interpolation.

**Table 1:** The Danish population and dominating influenza virus, by season

Season^1^	Dominant virus	Population (100,000)				
		
		All ages	0-4 years	5-14 years	15-64 years	Aged 65
1994/95	H3N2	52.18	3.35	5.67	35.17	7.99

1995/96	H3N2	52.50	3.43	5.77	35.35	7.96

1996/97	H3N2	52.74	3.46	5.90	35.45	7.93

1997/98	H3N2	52.95	3.46	6.06	35.51	7.92

1998/99	H3N2	53.13	3.44	6.23	35.55	7.91

1999/00	H3N2	53.30	3.41	6.40	35.59	7.91

2000/01	H1N1	53.49	3.38	6.56	35.63	7.92

2001/02	H3N2	53.68	3.35	6.69	35.68	7.95

2002/03	H3N2	53.83	3.32	6.80	35.72	7.99

2003/04	H3N2	53.97	3.30	6.87	35.76	8.05

2004/05	H3N2	54.12	3.28	6.90	35.81	8.13

2005/06	H3N2	54.28	3.25	6.90	35.89	8.23

2006/07	H3N2	54.48	3.25	6.89	35.99	8.36

2007/08	H1N1	54.75	3.26	6.84	35.12	8.53

2008/09	H3N2	55.09	3.27	6.81	36.26	8.76

2009/10	H1N1^2^	55.35	3.26	6.75	36.30	9.04

1) Season: week 27 to week 26 the following year. 2) Pandemic influenza A(H1N1) 2009/10

### Analyses

In our primary analysis, termed ILI-attributable mortality, we fitted a model with the weekly all cause number of death as outcome and ILI-activity and extreme temperatures as independent variables and adjusted for trend, seasonal variation, age and gender.

Influenza circulates simultaneously with other respiratory tract infections that may have been reported as ILI, especially in autumn and spring, i.e. in the beginning and end of the influenza season. To get an indication of the part of ILI associated with mortality associated with influenza we did a sub-analysis, where we reduced ILI in the beginning and end of the period with activity; based on the assumption that the major part of ILI will be influenza at the peak whereas influenza will not be a major contributor to ILI when ILI is low. This analysis was termed influenza-associated mortality.

### Statistical analyses

To estimate the association between weekly mortality, and influenza activity and extreme temperatures for the seasons 1994/5 to 2009/10 (week 27, 1994 to week 26, 2010), we used a multivariable time-series model with calendar week (wk) as underlying time unit, stratified by age groups (a = 0, 1-4, 5-14, 15-44, 45-64, 65-74, 75-84, 85+ years) and gender (g):

$$\text{E}\left(\text{M}{\text{R}}_{\text{a},\text{g},\text{wk}}\right)\;=\text{ E}\left({\text{D}}_{\text{a},\text{g},\text{wk}}∕{\text{N}}_{\text{a},\text{g},\text{wk}}\right)\;=\text{ E}\left({\text{D}}_{\text{a},\text{g},\text{wk}}\right)∕{\text{N}}_{\text{a},\text{g},\text{wk}}$$

where MR is the Mortality Rate, D number of all-cause deaths and N is the none-stochastic size of the population.

We used an additive Poisson regression model (link = id) with 1/population-size as offset parameter and allowing for overdispersion. For each age group and gender, the model for E(D) could formally be described as (omitting regression constants and parameters to be estimated):

$$\begin{array}{ccc} \text{E}\left(\text{D}\right) & =\text{spline}\left(\text{wk}\right)+ & \\ & \text{sin}\left(\text{2 }\pi \left(\text{365}.\text{25}∕\text{7}\right)*\text{wk}\right)\;+\text{ cos}\left(\text{2 }\pi \left(\text{365}.\text{25}∕\text{7}\right)*\text{wk}\right)\;+\\ & \text{sin}\left(\text{4 }\pi \left(\text{365}.\text{25}∕\text{7}\right)*\text{wk}\right)\;+\text{ cos}\left(\text{4 }\pi \left(\text{365}.\text{25}∕\text{7}\right)*\text{wk}\right)\;+\\ & \underset{\text{s}}{\sum }\underset{\text{wk}}{\sum }\text{IA }+\text{ w}{\text{c}}_{\text{wk}}+\text{ w}{\text{w}}_{\text{wk}}+\text{ s}{\text{c}}_{\text{wk}}+\text{ s}{\text{w}}_{\text{wk}}+\\ & \underset{\text{s}}{\sum }\underset{\text{wk}-\text{1}}{\sum }\text{IA }+\text{ w}{\text{c}}_{\text{wk}-\text{1}}+\text{ w}{\text{w}}_{\text{wk}-\text{1}}+\text{ s}{\text{c}}_{\text{wk}-\text{1}}+\text{ s}{\text{w}}_{\text{wk}-\text{1}}+\;\sum \text{epi} \end{array}$$

where the terms spline(wk) and the sine and cosine terms express the baseline with trend and seasonality. Trend was included as a cubic spline, and both a yearly and half-yearly seasonal cycles were included as sines and cosines. Impact of influenza activity (IA) may be heterogeneous between seasons [23]. Hence, IA was included separately for each season s (season: week 27 to week 26 the following year): ∑_s_∑_wk_IA. In the primary analysis IA was expressed as proportion of weekly ILI-consultations. Impacts of extreme temperatures were separated in weeks with extreme summer or winter temperatures, and include as the four variables: wc = winter cold, ww = winter warm, sc = summer cold and sw = summer warm (Figure 1). Further, deaths may be delayed relative to when IA was registered at a consultation or relative to when the temperature was extreme. Therefore, IA and extreme temperature event from the preceding week were also included. Finally, to compensate major unexplained peaks (outliers), residuals greater than 1.5 standard deviations, lasting 3 week or more and not explained by IA or extreme temperatures were included as artificial epidemic-parameters to compensate these (∑epi).

**Figure 2:**
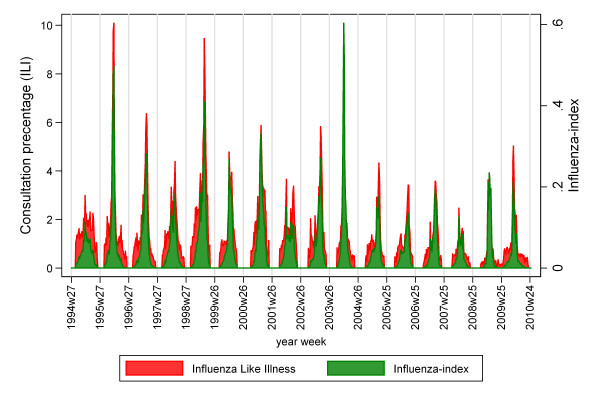
**Influenza like illness consultation percentage and the influenza-index**.

In order to obtain a more conservative estimation of influenza-associated mortality (rather than ILI), we applied an influenza-index to express influenza activity (IA), created by reducing the ILI consultation percentage for each season. This was done by multiplying the ILI consultation percentage in a specific season with a normal distribution, with mean and standard derivation as for the ILI percentage over the same season. This reduces the ILI percentage in the beginning and end of the season, and maintains the ILI percentage at the height of the season (Figure 2).

In the final model only trend and seasonal yearly or half-yearly cycles were included if they contributed on a 5% level (p < 0.05). Sensitivity to the temperature thresholds was investigated by varying the thresholds. Results are reported for the age groups 0-4, 5-14, 15-64 and 65+ years of age and for both sexes together. Hence, the results were adjusted for heterogeneities in pattern and variations between ages and genders. All analyses were done using Stata 11 MP.

## Results

**Figure 3:**
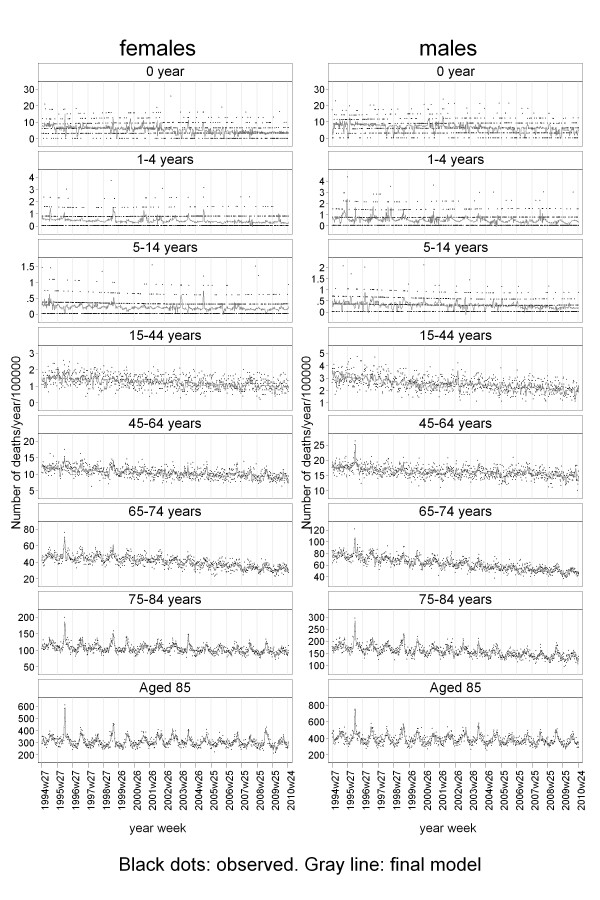
**Final model**.

The model fitted well in all age and gender strata (examined on deviances [24]), and there were no indications of either heteroskedasticity or residual autocorrelation (examined in correlograms). The results of the model are shown in Figure 3 for age groups and gender.

**Figure 4:**
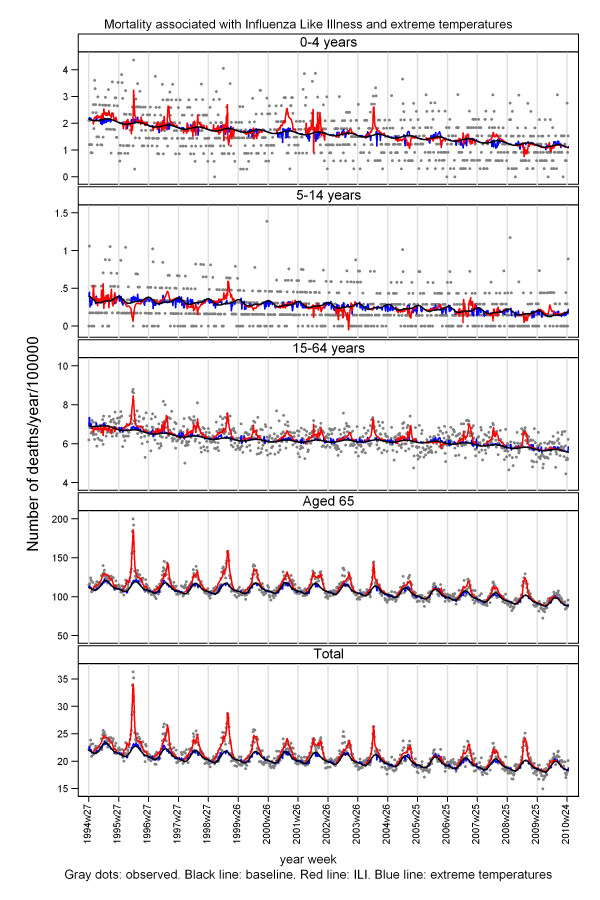
**Mortality associated with both ILI-activity and extreme temperatures**.

We found mortality to be associated with both ILI-activity and extreme temperatures (Figure 4).

The model included a baseline with both a yearly seasonal cycle and a half-yearly cycle, and the half-yearly cycle contributed on a 5% level in most strata. However, this was not due to a summer peaks; the half-year cycle created a seasonal pattern where the total seasonal cycle reaches its maximum earlier than for a symmetric yearly cycle, and as a consequence the decline became prolonged (baseline in Figure 4), corresponding more to the appearance of slightly asymmetric "epidemic curves" including a long right tail.

### ILI-attributable mortality

Over the seasons 1994/5 to 2009/10 the total median number of excess deaths per season attributable to ILI was 1879 (range: 306-5251) and the median mortality rate (MR) per 100,000 population was 35.4 (range: 5.6-100.0), table 2. Most of the ILI associated deaths per year was among elderly ≥ 65 years (median 87.9%) and adults 15-64 years (median 11.4%). There were nearly no ILI associated excess deaths among children (median 4 deaths for 0-4 years and -1 for 5-14 years of age), table 2.

**Table 2:** Excess mortality related to Influenza Like Illness, by season and age group

Season^1^	Death	Mortality^2^	Death	Mortality^2^	Death	Mortality^2^	Death	Mortality^2^	Death	Mortality^2^
**Age:**	**All ages**		**0-4 years**		**5-14 years**		**15-64 years**		**Aged 65**	

1994/95	1812	34.73 (31.80;37.65)	20	5.94 (2.90;8.99)	7	1.21 (0.36;2.05)	-185	-5.26 (-6.97;-3.54)	1970	246.72 (229.23;264.21)

1995/96	5252	100.04 (97.22;102.86)	10	2.88 (0.51;5.25)	-12	-2.16 (-2.83;-1.49)	531	15.04 (13.26;16.82)	4723	593.64 (576.84;610.43)

1996/97	2555	48.43 (45.69;51.17)	13	3.69 (1.19;6.20)	10	1.64 (0.85;2.44)	285	8.05 (6.22;9.88)	2247	283.27 (267.05;299.49)

1997/98	2287	43.20 (40.65;45.76)	4	1.28 (-0.89;3.46)	-9	-1.51 (-2.23;-0.78)	364	10.26 (8.76;11.76)	1928	243.52 (227.85;259.19)

1998/99	4477	84.26 (81.81;86.71)	-3	-0.73 (-2.95;1.49)	19	2.97 (2.23;3.71)	478	13.43 (12.01;14.85)	3983	503.84 (488.70;518.98)

1999/00	2125	39.87 (37.55;42.19)	-10	-2.99 (-5.10;-0.88)	-5	-0.73 (-1.41;-0.05)	220	6.18 (4.63;7.73)	1920	242.87 (228.89;256.85)

2000/01	1706	31.89 (29.19;34.58)	38	11.19 (8.58;13.80)	1	0.11 (-0.60;0.81)	220	6.17 (4.67;7.67)	1447	182.75 (165.90;199.61)

2001/02	2615	48.71 (46.34;51.09)	19	5.71 (3.43;7.99)	-5	-0.68 (-1.24;-0.12)	304	8.51 (7.09;9.93)	2296	288.98 (274.30;303.65)

2002/03	1672	31.06 (28.72;33.41)	3	0.98 (-1.24;3.21)	-13	-1.87 (-2.45;-1.30)	159	4.45 (3.04;5.87)	1523	190.66 (176.19;205.12)

2003/04	1946	36.05 (33.74;38.36)	21	6.36 (4.00;8.72)	0	-0.04 (-0.67;0.60)	287	8.02 (6.65;9.39)	1638	203.58 (189.39;217.77)

2004/05	1414	26.13 (23.60;28.66)	-15	-4.61 (-6.98;-2.23)	-6	-0.89 (-1.45;-0.33)	225	6.30 (4.76;7.83)	1210	148.85 (133.49;164.22)

2005/06	306	5.63 (3.06;8.20)	-6	-1.72 (-3.97;0.53)	5	0.74 (0.21;1.27)	-59	-1.65 (-3-29;-0.01)	365	44.35 (28.99;59.70)

2006/07	1718	33.37 (30.86;35.87)	0	0.03 (-2.32;2.37)	5	0.71 (0.09;1.32)	287	10.74 (9.17;12.32)	1426	170.70 (155.89;185.51)

2007/08	1486	27.13 (24.84;29.42)	14	4.16 (1.97;6.36)	-1	-0.13 (-0.69;0.44)	397	10.98 (9.67;12.30)	1076	126.12 (112.56;139.69)

2008/09	2113	38.35 (36.09;40.62)	-11	-3.42 (-5.48;-1.35)	-4	-0.65 (-1.22;-0.08)	291	8.02 (6.76;9.28)	1838	209.83 (196.61;223.06)

2009/10	541	9.77 (7.41;12.14)	-3	-0.85 (-2.79;1.08)	10	1.50 (0.85;2.15)	55	1.50 (0.02;2.99)	479	53.01 (39.84;66.18)

Median	1879	35.39	4	1.13	-1	-0.08	286	8.02	1738	206.71

1) Season: week 27 to week 26 the following year. 2) Deaths per 100,000 per year

Among adults, 15-64 years, there was an estimated annual number of ILI-associated deaths of 286 (range: -185 to 531) and the MR per 100,000 was 8.0 (range: -5.3 to 15.0). Among persons aged 65 the median number of deaths was 1738 (range: 365 to 4723) corresponding to a MR per 100,000 of 206.7 (range: 44.4 to 593.6). All estimates were adjusted for variations over age and between genders.

### Influenza-associated mortality

Not all reported ILI-cases were influenza. Hence, we reduced the ILI consultation percentage according to a pattern that reflected influenza-transmission (Figure 3). Compared to the total ILI-attributable number of deaths, this reduced the median total number of excess deaths with around 25% to 1420 (range: -8 to 3810) per season per 100,000, and MR to 26.4 (range: -0.1 to 72.6) (Table 3). Overall, we estimated that 82.0% of the influenza-associated deaths were among elderly aged 65 years.

**Table 3:** Excess mortality associated to Influenza expressed by the influenza-index, by season and age group

Season^1^	Death	Mortality^2^	Death	Mortality^2^	Death	Mortality^2^	Death	Mortality^2^	Death	Mortality^2^
**Age:**	**All ages**		**0-4 years**		**5-14 years**		**15-64 years**		**Aged 65**	

1994/95	877	16.82 (13.99;19.64)	-1	-0.22 (-2.96;2.51)	0	-0.00 (-0.81;0.80)	-233	-6.63 (-8.22;-5.03)	1111	139.16 (122.16;156.16)

1995/96	3810	72.58 (70.05;75.12)	7	2.14 (-0.15;4.43)	-10	-1.68 (-2.31;-1.05)	360	10.17 (8.72;11.62)	3453	434.03 (418.61;449.45)

1996/97	1827	34.64 (31.85;37.42)	3	0.74 (-1.72;3.19)	7	1.15 (0.37;1.92)	271	7.65 (5.92;9.39)	1546	194.95 (178.19;211.71)

1997/98	1432	27.04 (24.53;29.56)	0	-0.02 (-2.19;2.14)	-10	-1.60 (-2.30;-0.89)	276	7.77 (6.33;9.21)	1166	147.26 (131.79;162.73)

1998/99	3488	65.65 (63.23;68.08)	-11	-3.31 (-5.40;-1.22)	16	2.53 (1.77;3.28)	386	10.84 (9.45;12.24)	3098	391.91 (376.92;406.89)

1999/00	1802	33.80 (31.46;36.14)	-6	-1.83 (-3.94;0.28)	-3	-0.40 (-1.07;0.27)	158	4.43 (2.93;5.93)	1653	209.09 (194.87;223.31)

2000/01	1072	20.03 (17.32;22.74)	21	6.17 (2.77;9.57)	0	-0.03 (-0.74;0.67)	120	3.37 (1.92;4.82)	931	117.53 (100.49;134.57)

2001/02	2002	37.30 (34.99;39.62)	16	4.70 (2.47;6.93)	-2	-0.32 (0.87;0.24)	232	6.51 (5.18;7.83)	1756	221.02 (206.59;235.44)

2002/03	952	17.68 (15.46;19.90)	3	0.93 (-1.28;3.14)	-5	-0.74 (-1.43;-0.06)	122	3.41 (2.01;4.81)	832	104.20 (90.66;177.75)

2003/04	1409	26.10 (23.79;28.41)	17	5.12 (2.77;7.47)	1	0.18 (-0.43;0.79)	207	5.79 (4.45;7.13)	1183	147.07 (132.78;161.35)

2004/05	988	18.25 (15.66;20.84)	-13	-3.85 (-6.32;-1.37)	-7	-1.05 (-1.59;-0.52)	152	4.25 (2.69;5.81)	855	105.24 (89.47;212.01)

2005/06	-8	-0.14 (-2.68;2.40)	-6	-1.82 (-4.02;0.37)	5	0.68 (0.17;1.19)	-67	-1.86 (-3.59;-0.14)	61	7.37 (-7.60;22.33)

2006/07	1385	25.41 (22.93;27.90)	0	0.04 (-2.32;2.40)	3	0.48 (-0.17;1.13)	281	7.82 (6.31;9.33)	1100	131.60 (116.82;146.39)

2007/08	1465	26.76 (24.51;29.00)	17	5.20 (3.08;7.31)	-1	-0.21 (-0.75;0.34)	287	7.95 (6.66;9.24)	1162	136.23 (122.91;149.56)

2008/09	1744	31.65 (29.41;33.89)	-10	-3.19 (-5.23;-1.15)	-4	-0.57 (-1.13;-0.00)	215	5.92 (4.68;7.15)	1543	176.22 (163.11;189.33)

2009/10	121	2.19 (-0.19;4.57)	-2	-0.64 (-2-54;1.26)	8	1.17 (0.52;1.83)	-11	-0.29 (-1.72;1.14)	126	13.93 (0.58;27.29)

Median	1420	26.43	0	0.01	-1	-0.12	211	5.86	1164	143.11

1) Season: week 27 to week 26 the following year. 2) Deaths per 100,000 per year

### Temperature associated mortality

With our definition of a week having an extreme temperature, 38.5% of the weeks experienced extreme temperature (warm: 18.7%, cold: 19.8%) with a median extreme temperature of 1.4 (range: 0.3-4.1) degrees; warm: 1.5 (range: 0.4-4.1) degrees, cold: 1.3 (range: 0.3-3.2) degrees. Increasing the thresholds (minimum and maximum weekly temperatures) with up to 1 degree did not change the results substantially. Increasing the thresholds further reduced weeks with extreme temperatures seriously and therefore affected the results.

A yearly median of 39 deaths (range: -162 to 273) could be attributed to extreme ambient temperature, this corresponded to a MR per 100,000 of 0.7 (range: -3.0 to 5.2). These estimates were adjusted for variations over age and between genders (Table 4). The impact of extreme temperatures on mortality varied between benign (life saving) and malign (increased mortality) effects over the seasons, except among adults (15-64 years of age) where only malign effects of extreme temperatures were estimated. Further, a summer and winter differentiation in the impact of warm and cold temperatures was observed, with a benign effect of extreme cold during summer and the opposite in winter. For extreme warm temperatures it was the other way around (Table 5).

**Table 4:** Excess mortality associated to extreme ambient temperatures, by season and age group

Season^1^	Death	Mortality^2^	Death	Mortality^2^	Death	Mortality^2^	Death	Mortality^2^	Death	Mortality^2^
**Age:**	**All ages**		**0-4 years**		**5-14 years**		**15-64 years**		**Aged 65**	

1994/95	7	0.14 (-2.97;3.24)	-1	-0.19 (-3.88;3.49)	-2	-0.36 (-1.30;0.57)	50	1.42 (-0.48;3.32)	-40	-5.03 (-23.47;13.41)

1995/96	273	5.20 (1.88;8.52)	5	1.36 (-1.37;4.09)	0	0.07 (-0.61;0.74)	85	2.39 (0.22;4.57)	183	23.05 (3.43;42.67)

1996/97	86	1.63 (-1.54;4.80)	2	0.56 (-2.39;3.51)	-2	-0.34 (-1.27;0.58)	70	1.99 (-0.26;4.24)	16	1.99 (-16.46;20.44)

1997/98	117	2.22 (-0.70;5.13)	1	0.21 (-2.29;2.71)	-1	-0.24 (-1.09;0.61)	56	1.58 (-0.17;3.34)	62	7.80 (-9.98;25.59)

1998/99	112	2.28 (-0.49;5.06)	3	0.94 (-1.71;3.60)	-1	-0.17 (-1.09;0.74)	56	1.59 (-0.04;3.21)	63	7.93 (-9.16;25.02)

1999/00	-17	-0.32 (-2.91;2.26)	-1	-0.28 (-2.78;2.22)	-2	-0.34 (-1.14;0.46)	37	1.03 (-0.82;2.87)	-51	-6.40 (-21.66;8.85)

2000/01	-162	-3.02 (-6.18;0.13)	-2	-0.67 (-3.88;2.53)	-3	-0.45 (-1.31;0.41)	46	1.29 (-0.43;3.01)	-203	-25.57 (-45.35;-5.79)

2001/02	-139	-2.58 (-5.30;0.13)	-4	-1.13 (-3.95;1.69)	-2	-0.29 (-0.92;0.35)	32	0.90 (-0.80;2.59)	-165	-20.76 (-37.40;-4.12)

2002/03	241	4.47 (1.81;7.14)	4	1.13 (-1.55;3.81)	0	-0.05 (-0.69;0.59)	69	1.94 (0.29;3.60)	167	21.03 (4.72;37.33)

2003/04	64	1.19 (-1.30;3.68)	1	0.25 (-2.53;3.02)	-2	-0.26 (-1.00;0.48)	56	1.56 (0.09;3.04)	9	1.15 (-14.18;16.48)

2004/05	14	0.25 (-2.67;3.17)	2	0.52 (-2.30;3.34)	-3	-0.44 (-1.06;0.19)	62	1.72 (-0.04;3.48)	-47	-5.77 (-23.54;12.00)

2005/06	174	3.21 (0.13;6.29)	4	1.25 (-1.48;3.97)	0	-0.03 (-0.65;0.58)	74	2.05 (0.04;4.07)	97	11.77 (-6.50;30.04)

2006/07	-135	-2.47 (-5.32;0.38)	-5	-1.44 (-4.16;1.28)	-3	-0.39 (-1.08;0.30)	50	1.39 (-0.47;3.25)	-177	-21.22 (-37.93;-4.52)

2007/08	-146	-2.66 (-5.27;-0.05)	-3	-0.89 (-3.47;1.68)	-3	-0.39 (-1.04;0.26)	48	1.33 (-0.17;2.83)	-188	-22.06 (-37.51;-6.61)

2008/09	-8	-0.14 (-2.79;2.50)	0	-0.12 (-2.60;2.36)	-2	-0.23 (-0.89;043)	32	0.89 (-0.54;2.33)	-38	-4.39 (-19.91;11.13)

2009/10	164	2.97 (0.32;5.62)	2	0.66 (-1.50;2.81)	0	0.06 (-0.71;0.83)	61	1.67 (-0.01;3.36)	101	11.17 (-3.55;25.88)

Median	39	0.72	1	0.23	-2	-0.27	56	1.57	-15	-1.62

1) Season: week 27 to week 26 the following year. 2) Deaths per 100,000 per year

**Table 5:** Temperature associated mortality differentiated on summer and winter, all ages

Season^1^	Summer (week 14 to 39)						Winter (week 40 to 13)					
	
	Cold			Warm			Cold			Warm		
	
	Death	Mortality^2^	Weeks	Death	Mortality^2^	Weeks	Death	Mortality^2^	Weeks	Death	Mortality^2^	Weeks
1994/95	-30	-0.58 (-3.78;2.62)	7	67	1.29 (-1.87;4.45)	5	84	1.62 (-1.56;4.79)	4	-114	-2.19 (-5.38;1.00)	15

1995/96	-46	-0.88 (-4.23;2.48)	10	44	0.84 (-2.52;4.19)	6	313	5.97 (2.62;9.33)	17	-38	-0.73 (-4.09;2.62)	5

1996/97	-79	-1.50 (-4.74;1.75)	14	39	0.75 (-2.52;4.01)	6	199	3.78 (0.54;7.01)	12	-74	-1.40 (-4.66;1.87)	7

1997/98	-6	-0.11 (-3.11;2.88)	2	94	1.78 (-1.18;4.74)	11	116	2.19 (-0.78;5.15)	6	-87	-1.64 (-4.63;1.35)	7

1998/99	-65	-1.22 (-4.06;1.63)	15	-2	-0.03 (-2.89;2.82)	2	215	4.05 (1.22;6.87)	11	-28	-0.52 (-3.36;2.32)	5

1999/00	-22	-0.41 (-3.07;2.26)	3	72	1.36 (-1.26;3.98)	9	20	0.37 (2.30;3.04)	2	-88	-1.65 (-4.30;1.01)	9

2000/01	-87	-1.63 (-4.84;1.57)	15	0	0.00 (-3.24;3.24)	0	86	1.61 (1.62;4.83)	6	-160	-3.00 (-6.20;0.20)	14

2001/02	-19	-0.35 (-3.13;2.43)	6	50	0.94 (-1.83;3.71)	11	7	0.12 (-2.67;2.91)	1	177	-3.29 (-6.03;-0.56)	14

2002/03	-28	-0.51 (-3.23;2.21)	4	62	1.15 (-1.57;3.87)	9	235	4.36 (1.65;7.07)	15	-29	-0.53 (-3.27;2.21)	3

2003/04	-41	-0.76 (-3.36;1.84)	6	61	1.14 (-1.47;3.75)	9	124	2.30 (-0.29;4.89)	8	-80	-1.49 (-4.06;1.09)	6

2004/05	-105	-1.95 (-4.91;1.02)	15	20	0.37 (-2.64;3.38)	3	154	2.84 (-0.15;5.84)	9	-55	-1.01 (-4.01;1.98)	3

2005/06	-82	-1.51 (-4.60;1.58)	12	0	0.00 (-3.11;3.11)	0	286	5.27 (2.17;8.37)	14	-30	-0.55 (-3.65;2.56)	3

2006/07	-10	-0.18 (-3.11;2.76)	1	107	1.97 (-0.91;4.85)	15	0	0.00 (-2.94;2.94)	0	-232	-4.26 (-7.18;-1.35)	17

2007/08	-62	-1.14 (-3.78;1.51)	12	23	0.41 (-2.25;3.02)	5	83	1.51 (-1.15;4.17)	7	-189	-3.45 (-6.11;-0.79)	15

2008/09	-28	-0.51 (-3.21;2.18)	7	38	0.70 (-1.98;3.38)	6	53	0.96 (-1.73;3.65)	4	-71	-1.30 (-3.99;1.40)	10

2009/10	-22	-0.40 (-3.10;2.30)	7	56	1.02 (-1.67;3.70)	9	179	3.23 (0.05;5.91)	11	-49	-0.88 (-3.58;1.83)	6

Median	-36	-0.67	7	47	0.89	6	119	2.24	8	-77	-1	7

1) Season: week 27 to week 26 the following year. 2) Deaths per 100,000 per year

## Discussion

Over the seasons 1994/5 to 2009/10 we found a median all-cause mortality rate of 35.4 (range: 5.6-100.0) per season per 100,000 attributable to ILI for all ages and all inhabitants in Denmark, adjusted for trend, seasonality, extreme temperatures and heterogeneities between age groups and genders. Most of the mortality attributable to influenza was among the elderly (88%). In the younger age groups (< 65) there were seasons with an estimated benign impact of circulating influenza (negative mortality rates). This might have been due to few deaths and as a consequence low statistical power. However, some of the negative estimates had narrow confidence intervals below zero and cannot be ignored. We believe the benign impact of circulating influenza in the population is due to changes in care seeking behaviour (goes earlier to the doctor, stay home) due to knowledge of circulating influenza e.g. from the press.

The median all-cause mortality rate attributable to influenza for Denmark was within the range of estimates from Germany, USA, Italy and Canada based on all-cause mortality (Table 6) (range: 10 to 90 deaths per season per 100,000). However, it is important to underline that these estimates were obtained using different models and different periods.

**Table 6:** Mortality related to influenza

Country	Period	Age group	Mortality per 100,000	Method	Reference
West Germany Germany	1984/85-1994/95 1990/91-2000/01	All	16.1 (range: 2.2;35.7) 17.4 (range: 5.9;44.2)	All cause	Zucs 2005 [28]

USA	1972/73-2002/03	All	65.0 (range: 0;134.2) 72.4 (range11.7;144.7)	All cause	Thompson 2009 [3]

Canada	1997-2000 2000-2004	All	15,6 5.8 (vaccination)	All cause	Kwong 2008 [12]

Italy	1969-2001	All	23.4 (H3N2)/7.4 (H1N1)	All cause	Rizzo 2007 [6]

Canada	1989/90-1998/99	All	13.1	All cause	Schanzer 2007 [27]

South Africa USA	1998-2005 1997/98-2004/05	65+	340 112	All cause	Cohen 2010 [7]

Czech Republic	1982/83-1999/00	65+	26.0 (range: -8.9;60.4)	All cause	Kyncl 2005 [30]

Netherlands	1992-1996 1996-2003	65+	131 (95% CI: 123-139) 105 (95% CI: 99-111) (vaccination)	All cause	Jansen 2008 [31]

Hong Kong	1996-1999	65+	93.2 (95% CI: 78.4-107.7) 136.1 (95% CI: 83.7-188.4)	All cause	Wong 2004 [15]

Portugal	2008/09	65+	18	All cause	Nogueira 2009 [8]

Switzerland	1969/70-1998/99 1988/89-1998/99	60+	67.2 (range: 20.9;209,5) 152.8 (range: 66.3;293.3) 69.5 (range: 32.6;234,5) 187.1 (range: 121.0;356.0) 115.3 (range: 60.6;226.9)	All cause	Brinkhof 2006 [9]

Netherlands	1967-1989	60+	82	All cause	Sprenger 1993 [32]

USA	1976/77-2006/07	All	9.0 (range: 1.4;16.7) 2.4 (range: 0.4;5.1)	RC PI	MMWR 2010 [25]

Italy	1969-2001	All	4.5 (H3N2)/0.8 (H1N1)	PI	Rizzo 2007 [6]

USA France Australia	1972-1997	All	2.6 (range: 0;6.3) 3.9 (range: 0;18.3) 1.3 (range: 0;7.5)	PI	Viboud 2004 [4]

South Africa USA	1998-2005 1997/98-2004/05	65+	42 22	PI	Cohen 2010 [7]

Hong Kong	1996-1999	65+	16.7 (95% CI: 9.8-23.7) 39.3 (95% CI: 21.4-57.3)	PI	Wong 2004 [15]

France	1984-2004	65+	1.4 (range: 0;9.4)	PI	Denoeud 2007 [10]

RC: Respiratory and circulatory cause's incl. pneumonia and influenza. Vaccination: After introduction of vaccination programme

The most vulnerable age group for influenza death is the elderly with 80-90% of influenza related deaths among persons aged 65 [14,6,25,26,15]. We estimated the median Danish ILI-attributable mortality rate for persons aged 65 to 206.7 (range: 44.4 to 593.6), corresponding to 88% of all ILI deaths. Hence, our estimate of the all-cause mortality attributable to circulating influenza for persons aged 65 was within the range of estimates from other countries (table 6).

Surveillance of ILI, will include respiratory infections caused by other pathogens than influenza virus that may be perceived as influenza-like illness. Hence, excess mortality attributable to circulating influenza will over-estimate mortality associated to influenza. Therefore, PI as cause of death has been used to estimate influenza-associated mortality [25,6,4,15,10]. Using PI-specific mortality has been found to reduce mortality attributable to circulating influenza with 38% among Swiss elderly (60+) [9], and 40% for all ages in Canada [27] compared to estimates based on all-cause mortality. However, for various reasons, estimates based on PI as cause of death may tend to under-estimate influenza-associated mortality. Death due to influenza, or where influenza was an important component in the chain of events that lead to the death, may not be coded as respiratory, but rather as cardiac or related to other complications of severe illness.

We estimated mortality attributable to ILI directly as a parameter in our model. To obtain estimates of influenza-associated mortality, we could have used data from laboratory based surveillance instead of ILI. However, the virological surveillance of influenza in Denmark is not sufficiently systematic and detailed to be used directly as a parameter to model influenza-associated mortality. Although samples are collected by the sentinel practitioners, they usually collect only three times in a season, which means that the samples do not reflect the amount of circulating influenza, i.e. are not suitable for modelling. Likewise, data on clinical samples from hospitals are, except for the 2009/10 pandemic, few in numbers for many seasons. We therefore used a proxy to downgrade ILI to influenza as described above, and estimated an all-cause influenza-associated mortality of 26.4 (range: -0.1 to 72.6) per season per 100,000, corresponding to a 25% lower mortality compared to ILI-estimates, and slightly higher than PI-associated mortality reported from other countries (table 6). Therefore, with mortality attributable to ILI being an over-estimate and PI-associated mortality probably an under-estimate our estimate, using the influenza-index, may well be a realistic estimate of influenza-associated mortality.

**Figure 5:**
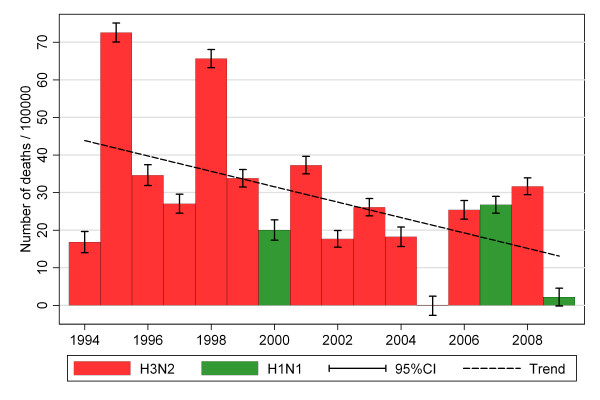
**Influenza(ILI)-attributable mortality, according to dominant type of influenza**.

Mortality related to influenza has been found to be higher in season where the dominating influenza is H3N2, compared to H1N1 seasons [5,28,7,25]. We found particular high excess mortality in the two influenza A(H3N2) seasons of 1995/96 and 1998/99, whereas the influenza A(H3N2) season of 2005/6 and the 2009 influenza A(H1N1) pandemic had none or only modest impact on mortality. In addition, there has been a declining trend in influenza related mortality since the 1990's (Figure 5). This trend remains even after excluding the low-mortality 2005/6 H3N2 season and the 2009/10 H1N1 pandemic, although not that strong. The reasons for this trend are not obvious, but increased use of vaccines for the elderly, genetic drift of the virus as well as herd immunity in the population may contribute.

Over the seasons 1994/5 to 2009/10 we found an all-cause median mortality rate of 0.7 (range: -3.0 to 5.2) per season per 100,000 attributable to periods with extreme temperatures for all ages and all inhabitants in Denmark, adjusted for trend, seasonality, influenza activity and heterogeneities between age groups and genders. However, this consists of both periods with extreme cold and heat, and covers both summer and winter. Perhaps not surprisingly, there seems to be a pattern where periods with cold weather in the summer is life saving, while heat may cost lives. In the winter season warm periods saves lives, while cold cost lives.

We have proposed and evaluated a methodology and statistical model usable to estimate excess mortality associated to two potential explanatory factors, ILI and extreme temperatures based on all-cause mortality. The model fitted data well and all-cause mortality has previously been found to be the most complete and accurate in assessing the total impact of influenza on mortality [29]. More explanatory factors may be included in the model like for example infection with RSV [11], other cause of acute respiratory illnesses and invasive pneumococcal disease, to get more detailed estimates of variations in excess mortality. A limitation of the model is potential interactions between the explanatory factors, which cannot be easily implemented in the model; especially with more than two explanatory factors. As influenza virus circulates simultaneously with other respiratory infections, ILI may include infections caused by other pathogens than influenza virus and may as such be an overestimation of circulating influenza. Denmark does not have systematic ongoing laboratory surveillance of influenza, but among influenza-samples received at the reference laboratory at Statens Serum Institut, the proportion of influenza positive samples is typically low in the beginning and end of the season, and highest around the peak of ILI. Our influenza-index reflects this pattern Others have used PI cause-specific mortality as outcome, and our methodology gives influenza-associated mortality that falls between the over-estimation using all cause mortality and the under-estimation using PI cause specific mortality. Further, it has the advantage of depending only on easily obtainable data namely weekly number of all-cause deaths, temperature, and ILI.

## Conclusions

We have shown that it is doable to model seasonally fluctuations in mortality related to ILI or influenza and to extreme ambient temperatures based on all-cause mortality. This is promising, as all-cause data is easier to obtain than cause-specific data, and not subject to coding bias. It is recognised that coding practices will be affected by awareness and media reports. However, it is of interest to apply our methodology to other datasets in order to validate it further. We also advocate that laboratory based influenza surveillance should be reinforced in order to better estimate the proportion of severe respiratory illness that are caused by influenza. This may serve as a more appropriate independent variable in future multivariable models. Finally, it is worth noting that influenza mortality has tended to decline and studies of the effect of in particular influenza vaccination policies on this trend are needed.

## Competing interests

The authors declare that they have no competing interests.

## Authors' contributions

JN did the data analyses and was the lead writer. AM, SG and KM wrote part of introduction and discussion, and contributed with ideas and comments to methods and results. All authors read and approved the final manuscript.

## Pre-publication history

The pre-publication history for this paper can be accessed here:

http://www.biomedcentral.com/1471-2334/11/350/prepub
